# Early Diagnosis to Improve the Poor Prognosis of Pancreatic Cancer

**DOI:** 10.3390/cancers10020048

**Published:** 2018-02-11

**Authors:** Masataka Kikuyama, Terumi Kamisawa, Sawako Kuruma, Kazuro Chiba, Shinya Kawaguchi, Shuzo Terada, Tatsunori Satoh

**Affiliations:** 1 Department of Gastroenterology, Tokyo Metropolitan Cancer and Infectious Disease Center Komagome Hospital, 3-18-22, Honkomagome, Bunkyo-ku, Tokyo 113-8677, Japan; kamisawa@cick.jp (T.K.); sawako@cick.jp (S.K.); kazuro_oruzak@yahoo.co.jp (K.C.); 2 Department of Gastroenterology, Shizuoka General Hospital, Shizuoka 420-8527, Japan; shinya-kawaguchi@i.shizuoka-pho.jp (S.K.); m01060st@jichi.ac.jp (S.T.); tatsunori-sato@i.shizuoka-pho.jp (T.S.)

**Keywords:** EUS, ERCP, SPACE, MDCT, MRI, FDG-PET, DM, acute pancreatitis, hereditary pancreatitis, carcinoma in situ

## Abstract

Pancreatic cancer (PC) has a poor prognosis due to delayed diagnosis. Early diagnosis is the most important factor for improving prognosis. For early diagnosis of PC, patients with clinical manifestations suggestive of PC and high risk for developing PC need to be selected for examinations for PC. Signs suggestive of PC (e.g., symptoms, diabetes mellitus, acute pancreatitis, or abnormal results of blood examinations) should not be missed, and the details of risks for PC (e.g., familial history of PC, intraductal mucin producing neoplasm, chronic pancreatitis, hereditary pancreatitis, or life habit) should be understood. Multidetector computed tomography (MDCT) and magnetic resonance imaging (MRI) can be performed for diagnosing PC, but the diagnostic ability of these examinations for PC is limited. Endoscopic diagnostic procedures, such as endoscopic ultrasonography, including fine-needle aspiration, and endoscopic retrograde pancreatocholangiography, including Serial Pancreatic-juice Aspiration Cytologic Examination (SPACE), could be recommended for a detailed examination to diagnose pancreatic carcinoma earlier.

## 1. Introduction

Pancreatic cancer (PC) has a poor prognosis. The National Cancer Center Japan estimated that 39,800 individuals had PC, and 34,100 individuals died because of PC in 2017 in Japan, and the five-year survival rate for PC is 7.8%. PC is the fourth-most common cause of cancer-related deaths among men and women in Japan. The poor prognosis related to PC is due to delayed diagnosis, because >90% of patients with PC are diagnosed at stages III and IV [1]. 

Early diagnosis is required to improve prognosis, and the clinical manifestations suggestive of PC need to be understood for early diagnosis and examining those who are at high risk for developing PC. An appropriate imaging technique should be selected to diagnose PC.

## 2. Clinical Manifestations Suggesting PC

Clinical manifestations suggestive of PC have been reported, and the information can provide the indication for selecting patients who need to undergo further diagnostic workup for PC.

### 2.1. Symptoms

The Pancreatic Cancer Registry Report of Japan 2007 [2] described that patients with PC had symptoms related to PC, except for 15.4%. Abdominal pain was the most common symptom, with 31.6% of patients experiencing this symptom (Table 1). Jaundice and back pain also occurred in 18.9% and 8.6% of patients, respectively. Among patients with small PC (diameter, <2 cm), 36.8% were asymptomatic. However, 24.5% of patients with small PC had abdominal pain, which was the most common symptom.

A recent report on the characteristics of PC resected at an early stage was published [3]. Fifty-one and 149 patients with stage 0 and I PC, respectively, were estimated (Table 2). Of these patients, 50 (25.0%), 103 (51.5%), 34 (17.0%), and 15 (7.5%) visited a hospital because of symptoms, abnormal findings indicative of other disorders, abnormal results of screening and medical check-up, and other, respectively. Because evaluation was limited to an early stage, symptomatic patients were few. Abdominal pain was the most common symptom, which accounted for 72% of all symptoms, followed by back pain, nausea, diarrhea, and jaundice in 13 (26.0%), 4 (8.0), 1 (2.0), and 1 (2.0) patients, respectively.

PC has no specific symptoms. However, abdominal pain could suggest PC, and those with abdominal pain should be evaluated for PC.

### 2.2. Diabetes Mellitus 

Diabetes mellitus (DM) was present in 24% of patients with PC [2]. A meta-analysis showed that the risk for PC in patients with DM was twice that in those without DM [4]. Furthermore, PC was most frequently diagnosed within 1 year after DM onset [4]. Other studies described that 0.25% of patients had PC within 3 years after DM onset, and the frequency was twice that in the control [5]. For patients older than 50 years with DM, 1% had PC within 3 years after DM onset [6]. On the other hand, 48 (29.3%) patients of 187 patients with PC had DM, and DM was diagnosed in 21 (52.5%) and 14 (35%) of 40 patients with PC within 2 and 5 years after PC diagnosis, respectively [7]. Acute exacerbation of DM could also be a sign of PC, accounting for approximately 5% of patients with PC [2].

In patients with DM, age older than 65 years, smoking, history of gallstone disease, chronic pancreatitis, and non-obesity were proposed as factors associated with PC [5]. Moreover, endoscopic retrograde cholangiopancreatography (ERCP) helped diagnose PC in 13.9% of patients with new-onset DM who were over 55 years old, had increased pancreatic enzyme or tumor marker level, or had abnormal finding in the pancreas on ultrasonography [8].

New-onset DM and acute exacerbation of DM can be signs of PC, and a detailed examination is needed. Factors associated with PC also can be helpful in selecting patients who should undergo a detailed examination.

### 2.3. Acute Pancreatitis

In patients with acute pancreatitis, 6.8% had PC within 2 years after the onset of acute pancreatitis, and one third of the tumors were small PCs [9]. Another study reported that 1.45% of patients with acute pancreatitis developed PC within the same period, and the incidence of PC reduced in the third year; further, age older than 40 years was an added risk factor for PC [10]. Cases of carcinoma in situ were recently reported to be the cause of acute pancreatitis [11]. 

Acute pancreatitis can be an indicator of PC, and patients with acute pancreatitis should be observed for 2 years using diagnostic imaging techniques.

### 2.4. Abnormal Results of Blood Examination: Tumor Marker

Various types of tumor markers are used for diagnosing PC. Particularly, Carbohydrate antigen 19-9 (CA19-9) is superior because of its high positive sensitivity of 70.9%, with a sensitivity of 52.3% for small PC [2]. When the reference value of CA19-9 was accepted as 74 U/mL, the specificity was 100% when pancreatic carcinoma was compared with benign disorders of the pancreas [12]; that is to say, CA19-9 can contribute to differentiating PC from benign lesions [13]. However, we should be reminded that cholestasis induces a pseudo-positive result for PC, and a negative Lewis antigen result is responsible for a false-negative result for CA19-9. In patients with DM, Carcinoembryonic antigen (CEA) >5 ng/mL or CA19-9 >500 U/mL is suggestive of PC [14].

Recently, a novel tumor marker, thrombospondin-2 (THBS-2), has been produced. A THBS-2 and CA19-9 panel assessed in human blood using a conventional ELISA assay may improve the detection of patients as high risk for PC [15].

CA19-9 level provides valuable information with which to suspect PC. In patients with DM, CA19-9 is also a preferable indicator for selecting candidates for detailed examination.

### 2.5. Abnormal Results of Blood Examination: Pancreatic Enzyme

Abnormal results on blood examinations for pancreatic enzymes were recognized in one-third of patients with PC [2]. Various disorders, such as pancreatitis, renal dysfunction, and macro-amylasemia, could cause increased pancreatic enzyme levels; hence, increased pancreatic enzyme level is not specific for PC. If patients have increased pancreatic enzyme levels, imaging should be performed to determine whether the dilation of the pancreatic duct is due to PC.

## 3. PC High-Risk Group

Diseases considered to increase the risk of developing PC have been identified, and imaging should be performed at a regular interval of 6 months to diagnose PC earlier although patients are asymptomatic.

### 3.1. Familial History of PC

A family history of PC is observed in 5% to 10% of patients with PC. Gene mutations in *CDKN2A(p16)*, *BRCA2*, and *PALB2* are considered to be associated with PC. Familial PC is defined as having two or more first-degree relatives with PC. The relative risk for PC is 2.41 in sporadic cases (i.e., families with only one relative with PC or with multiple PCs in more distant relatives and/or spouses with PC), whereas the risk increases to 6.79 and 17.2 times in cases with two and more first-degree relatives with PC, respectively [16]. In the familial PC kindreds, risk varied by the number of first-degree relatives with PC, such that risk was higher in individuals with three first-degree relatives who had PC (Standardized Incidence Ratio, SIR = 17.02; 95% CI = 7.34 to 33.5; *p* < 0.01), but lower in individuals who had two first-degree relatives with PC (SIR = 3.97, 95% CI = 1.59 to 8.2, *p* = 0.05) or with one affected first-degree relative (SIR = 6.86, 95% CI = 3.75 to 11.04, *p* < 0.001). Whereas risk was higher for familial PC kindred members who had one first-degree relative with PC as compared with two, the confidence intervals for these two estimates largely overlap [16].

A familial history of PC suggests a high risk for PC, and the incidence of PC depends of the number of first-degree relative with PC. Identification of a family history of PC is important.

### 3.2. Intraductal Mucin Producing Neoplasm (IPMN)

PC is well known to be accompanied by IPMN. The incidence of PC was reported to be 2% in 349 patients with IPMN who were observed for 3.7 years [17]. Another study reported that PC occurred in 5 of 60 (8%) patients with IPMN (with a diameter <10 mm), who were observed for 87 months, and the 1- and 5-year morbidity rates were 1.1% and 6.9%, respectively [18]. Moreover, an age of 70 years and older was a risk factor for PC accompanied by IPMN [18]. The presence of any pancreatic cyst, including IPMN, was reported to be a high-risk factor for PC development, accounting for 0.95% of patients with pancreatic cysts per 1 year, which was 22.5 times higher than that in individuals without pancreatic cystic lesions [19].

IPMN should be carefully observed because of the high incidence of PC, and the presence of any pancreatic cyst is a high-risk factor for PC.

### 3.3. Chronic Pancreatitis

In chronic pancreatitis, >2-year observation showed that the relative risk for PC in chronic pancreatitis was 16.5 to 26.7, and the incidence ratios of PC occurrence were reported to be 1.1%, 1.8%, and 4.0% in 5-, 10- and 20-year observations, respectively [20,21]. 

### 3.4. Hereditary Pancreatitis

Hereditary pancreatitis is defined by the following: acute recurrent pancreatitis or chronic pancreatitis in two and more members of a family; an absence of a history of alcohol abuse in at least one patient; and pancreatitis in at least one brother or sister younger than 40 years. If the patient has a *p.R122H* or *p.N291* mutation on *PRSS1*, the diagnosis of hereditary pancreatitis is confirmed, irrespective of the definition. In hereditary pancreatitis, the relative risk for PC is 50, and the proportions of patients developing PC due to hereditary pancreatitis are 10%, 18.7%, and 53.5% in 50-, 60-, and 75-year-old patients, respectively [22].

Familial history of pancreatitis should be considered when examining a patient with pancreatitis.

### 3.5. Life Habit

Smoking and alcohol abuse are associated with PC [23,24]. In particular, smoking increases the risk for PC, contributing to 2–35% of PC cases [25,26,27]. In current smoking, the relative risk (RR) of PC development was reported to be 1.74 [25], and the risk remains, with a RR of 1.48 even after 10 years smoking cessation [25]. The association of alcohol intake and PC has been recognized in a dose-dependent manner [28]. The association was slight, with a RR of 1.22 for alcohol intake greater than 30 g/day, while the association was stronger, with a RR of 1.53 when the consumed alcohol was greater than 60 g/day. The association of cigarette smoking with PC occurrence is thought be due to the release of various carcinogenetic agents from cigarettes. 

Dietary habits including the consumption of red meat and processed meat increased the risk of PC, with a RR of 1.29 in men and 1.19 in both men and women, respectively [29]. Body mass index (BMI) indicated the risk of PC; a 5 kg/m^2^ increase in BMI was associated with a 12% increased risk of PC [30].

In Japan, the positive association of smoking with PC was recognized with a RR of 3.3 [31]. Moreover, the association of smoking and other life-style factors with PC death was examined [32]. The results showed that a positive association between smoking and death from pancreatic cancer in Japanese women (Hazard ratio: 4.77), but not in men, while the associations of the other lifestyle factors including alcohol or diet were equivocal in men and women [32]. However, this study has the limitations of a lack of statistical power and the use of mortality data instead of incidence data.

## 4. Diagnostic Imaging Techniques

The factors described previously can provide information for suspecting PC and for selecting patients as candidates for detailed examination using imaging techniques. Generally, multidetector computed tomography (MDCT) and magnetic resonance imaging (MRI) are recommended for diagnosing PC, and these imaging techniques are widely used when PC is suspected. However, characteristics of various diagnostic imaging techniques, including MDCT and MRI, should be understood to diagnose PC appropriately.

### 4.1. MDCT

MDCT is the most commonly selected imaging technique in diagnosing PC. Its sensitivity rate for PC was reported to be 89–97% [33]. However, this imaging technique is limited to detecting PC <20 mm because 23% of small PC cannot be detected [34]. Moreover, MDCT cannot reveal tumors in 33% of PC <15 mm [35]. This low sensitivity for detecting tumors may be because of not only the size, but also the pathological characteristics of the tumor, with lower tumor cellularity, more frequent intratumoral acinar tissues and islet cells, and less prominent tumor necrosis [36]. 

One should remember the limited ability of MDCT in detecting tumors in small PC. If secondary signs suggesting PC, such as pancreatic duct dilation, are detected by MDCT [35,36], or clinical manifestations are presented by the patient, leading to suspicion of PC, other imaging techniques should be added for further examination.

### 4.2. MRI

An enhancing pattern of PC on MRI is similar to that on MDCT, but stronger contrast enhancement and superior ability to detect PC are advantages of MRI compared with MDCT [37]. MRI can identify 80% of PCs that were not recognized by MDCT [36]. Recently, the value of diffusion-weighted MRI (DWI) has been recognized in diagnosing PC [38]. DWI can help detect solid pancreatic neoplasms with extremely dense cellularity or extracellular fibrosis by demonstrating significantly low apparent diffusion coefficient (ADC) values [39]. However, DWI may not be capable of definitively characterizing solid lesions as inflammatory or neoplastic because of an overlap in ADC values between the two types [39]. 

MRI contributes to detecting pancreatic lesions that cannot be identified by MDCT; however, another diagnostic method is needed to identify the characteristics of the lesion. 

### 4.3. Positron Emission Tomography with 2-Deoxy-2-[fluorine-18]fluoro-d-glucose (FDG-PET)

The sensitivity and specificity of FDG-PET (2-Deoxy-2-[fluorine-18]fluoro-d-glucose) for PC are 89% and 88%, respectively, which are superior to those of MDCT and MRI [40]. Moreover, FDG-PET is a better diagnostic technique for detecting PC < 20 mm than MDCT and MRI [41].

FDG-PET is widely used for cancer staging or revealing recurrent lesions, and advancement of the role of FDG-PET in the clinical stage is expected.

## 5. Diagnostic Endoscopic Technique

The diagnostic imaging techniques described above have been advanced, and are widely used in diagnosing PC. However, almost all PCs are diagnosed late, leading to poor prognosis. To improve the prognosis of PC, early diagnosis is very important. Endoscopic ultrasonography (EUS) has recently become a widely used method for diagnosing PC, and several studies have reported its superiority. Moreover, a recently developed method using ERCP is expected to diagnose PC earlier.

### 5.1. EUS

EUS is an invasive examination. However, its superiority for diagnosing PC has been reported [42], especially in comparison with MDCT [43]. The highest negative predictive value among imaging techniques for PC was recognized [44]. EUS is particularly superior for detecting solid lesions, such as PC, whereas MRI was superior for detecting cystic lesions [45]. 

EUS has been used for a detailed examination of lesions recognized by computed tomography (CT) or MRI. However, EUS has become the most sensitive examination for PC in patients with clinical manifestations suggesting PC or high-risk factors for PC. Moreover, EUS-guided fine needle aspiration can confirm the diagnosis of PC by puncturing pancreatic lesions through the gastrointestinal tract wall [46].

### 5.2. ERCP

ERCP has been used not only for performing pancreatography, but also for diagnosing PC, using pancreatic duct brushing for cytology. However, the sensitivity of cytology for diagnosing PC is low [47]. Moreover, post-ERCP pancreatitis is the most troublesome adverse event. ERCP has been considered to be inadequate for examining PC.

Serial pancreatic juice aspiration cytologic examination (SPACE), a new diagnostic method using a nasopancreatic tube placed via the major papilla by ERCP, was recently developed [48]. It was able to provide high sensitivity and specificity, even for a small lesion or pancreatic carcinoma in situ (PCIS) [48]. 

EUS is a prominent procedure in diagnosing small PC. However, EUS does not work if the tumor is too small or faint to be recognized by EUS. PC originates from the pancreatic duct mucosa, and PC forms a tumor invading the surrounding tissues. Even if PC is diagnosed at an early stage, the tumor is invasive and advanced. If PC is diagnosed at the stage of PCIS before forming a tumor, no metastasis will occur, and excellent treatment for PC is expected. SPACE can provide the opportunity to diagnose PCIS at its earliest stage, stage 0, before invasion of the surrounding tissues.

The candidates for SPACE are patients with pancreatic duct strictures. The occurrence of pancreatic duct strictures is due to periductal fibrosis, which is induced by chronic inflammation. In some cases, PCIS induces fibrosis via local pancreatitis or secretion of some cytokines, such as transforming growth factor-β [49]. PCIS should be considered if a pancreatic duct stricture without a tumor is seen adjacent to the stricture, particularly with fat replacement on CT or hypoechoic area on EUS around the stricture [11]. 

## 6. Diagnostic Strategy for PC

For early diagnosis of PC, patients with clinical manifestations suggestive of PC and high risk for developing PC need to be selected for examinations for PC. Although MDCT and MRI could be performed for diagnosing PC, the diagnostic ability of these examinations for PC is limited, and EUS should be considered for detailed examination. If patients have a pancreatic duct stricture, ERCP for SPACE is recommended, despite there being no tumor related with the stricture. 

## 7. Conclusions

Signs suggestive of PC (e.g., symptoms, diabetes mellitus, acute pancreatitis, or abnormal results of blood examinations) should not be missed, and the details of the risks for PC (e.g., familial history of PC, intraductal mucin producing neoplasm, chronic pancreatitis, hereditary pancreatitis, or life habit) should be understood in order to diagnose PC at an early stage to improve its prognosis. For diagnosing smaller or earlier PC, appropriate diagnostic techniques, including EUS and SPACE using ERCP, should be selected.

## Figures and Tables

**Table 1:** The Pancreatic Cancer Registry Report of Japan 2007.

Symptoms Related to Pancreatic Cancer	Pancreatic Cancer (%)	Small Pancreatic Cancer (%)
Symptoms	84.6	63.2
Abdominal Pain	31.6	24.5
Jaundice	18.9	
Back Pain	8.6	

**Table 2:** Occasion of visiting a hospital in resected pancreatic cancer at an early stage (Stage 0/I = 49/151).

Reasons to Visit a Hospital	N (%)
Symptoms	50 (25.0)
Abdominal pain	36 (72.0)
Back pain	13 (26.0)
Nausea	4 (8.0)
Diarrhea	1 (2.0)
Jaundice	1 (2.0)
Abnormal findings indicative of other disorders	103 (51.5)
Abnormal results of screening and medical check-up	34 (17.0)
Others	15 (7.5)
